# Paclitaxel and carboplatin *vs* gemcitabine and vinorelbine in patients with adeno- or undifferentiated carcinoma of unknown primary: a randomised prospective phase II trial

**DOI:** 10.1038/sj.bjc.6604818

**Published:** 2008-12-09

**Authors:** G Huebner, H Link, C H Kohne, M Stahl, A Kretzschmar, S Steinbach, G Folprecht, H Bernhard, S E Al-Batran, P Schoffski, C Burkart, F Kullmann, B Otremba, M Menges, M Hoffmann, U Kaiser, A Aldaoud, A Jahn

**Affiliations:** 1 Westpfalz-Klinikum, Hellmut-Hartert-Str 1, 67655 Kaiserslautern, Germany; 2 University Hospital Carl Gustav Carus, Fetscherstr 74, 01307, Dresden, Germany; 3 Kliniken Essen-Mitte, Henricistr 92, 45136 Essen, Germany; 4 Helios-Klinikum Berlin-Buch, Lindenberger Weg 80, 13125 Berlin, Germany; 5 University Hospital rechts der Isar, Ismaninger Str 22, 81675 Munich, Germany; 6 Klinikum Frankfurt-Nordwest, Steinbacher Hohl 2-26, 60488 Frankfurt, Germany; 7 Hanover Medical School, Carl-Neuberg-Str 1, 30623 Hannover, Germany; 8 Eberhard Karls University Hospital, Ottfried-Müller-Str 1, 72076 Tubingen, Germany; 9 University Hospital, Franz-Josef-Strauß-Allee 11, 93042 Regensburg, Germany; 10 Oncology Practice, Auguststr 22, 25121 Oldenburg, Germany; 11 Saarland University Hospital, Kirrberger Str 100, 66421 Homburg, Germany; 12 Klinikum Ludwigshafen, Bremser Str 79, 67063 Ludwigshafen, Germany; 13 St. Bernward Hospital, Treibestr 9, 31334 Hildesheim, Germany; 14 Oncology Practice, Strümpelstr 41, 04289 Leipzig, Germany; 15 Johannes Gutenberg University, Langenbeckstraße 2, 55131 Mainz, Germany

**Keywords:** neoplasms, unknown primary, randomized controlled trial, clinical trial, phase II, medical oncology, anti-neoplastic-combined chemotherapy protocols

## Abstract

Platinum/taxane combinations are widely used in patients with carcinoma of unknown primary (CUP), yielding response rates of 30% and median overall survival of 9–11 months in selected patients. Yet these combinations have not been subject to a randomised trial to overcome selection bias, a major problem in CUP. We randomised 92 patients to either paclitaxel/carboplatin (arm A) or the non-platinum non-taxane regimen gemcitabine/vinorelbine (arm B). The primary endpoint was rate of practicability as defined: application of ⩾2 cycles of therapy (1) with a maximal delay of 1 week (2) and survival of ⩾8 months (3). Practicability was shown in 52.4% (95% CI 36–68%) in arm A and in 42.2% (95% CI 28–58%) in arm B, respectively. The median overall survival, 1-year survival -rate and response rate of patients treated in arm A was 11.0 months, 38, and 23.8%, arm B 7.0 months, 29, and 20%. In conclusion, the paclitaxel/carboplatin regimen showed clinically meaningful activity in this randomised trial (Clinical trial registration number 219, ‘Deutsches KrebsStudienRegister’, German Cancer Society.)

Despite improved diagnostic tools (e.g., imaging procedures and histologic/molecular workup), carcinoma of unknown primary site (CUP) remains a common clinical problem and represents 2–4% of all malignancies (Levi *et al*, 2002; van de Wouw *et al*, 2002). Histologically, adenocarcinoma (40–50%) and poorly differentiated carcinoma including poorly differentiated adenocarcinoma (30–40%) account for the vast majority of cases. In recent years comparable clinical behaviour of both groups was demonstrated and ‘treatable’ subsets were identified: resectable disease, squamous or poorly differentiated carcinoma in upper cervical nodes, women with axillary nodes (suggestive of occult breast cancer), women with peritoneal carcinomatosis (suggestive of occult ovarian cancer), young men with features of extragonadal germ cell cancer (young age, poorly differentiated carcinoma, midline presentation, multiple pulmonary nodules), neuroendocrine carcinoma, and possibly colon-cancer-type carcinoma (Varadhachary *et al*, 2008). Unfortunately, these subsets represent only 5–10% of cases (Pavlidis and Fizazi, 2005). In the remaining patients improved results with recent empiric therapies incorporating new chemotherapeutic agents have replaced the former attitude of widespread nihilism in the majority of oncologists.

Platinum/taxane combinations are widely used in patients with CUP, yielding response rates of about 30% and a median survival of 9–11 months in selected patients in large phase II studies (Hainsworth *et al*, 1997; Greco *et al*, 2002). Gemcitabine has shown efficacy in second-line treatment and has recently been incorporated in first-line regimen. Combined with cisplatin the response rate was 55% with a median survival of 8 months (Culine *et al*, 2003). The combination with carboplatin resulted in a 31% response rate with equal survival (Pittman *et al*, 2006). Gemcitabine in combination with docetaxel yielded a response rate of 40% with a median survival of 10 months (Pouessel *et al*, 2004). Vinorelbine has proven efficacy in many malignancies, especially non-small cell lung cancer (NSCLC) and breast cancer, with only anecdotal experience in CUP syndrome.

To date only eight randomised trials have been published in CUP syndrome, none of these addressing the efficacy of platinum/taxane combinations (Fizazi and Schmoll, 2006). This study was designed to investigate carboplatin/paclitaxel and the non-platinum non-taxane regimen gemcitabine/vinorelbine in a prospective randomised multicenter phase II trial.

## Patients and methods

Patients with histologically/cytologically proven adeno- or undifferentiated metastatic CUP site were eligible for participation in this multicenter study. Complete history, physical examination, chest X-ray, CT scan of chest and abdomen, upper intestinoscopy, colonoscopy, mammography (in women), PSA, AFP, and hCG (in men), and directed workup of symptomatic areas were mandatory before inclusion. Patients with resectable disease, men with features of the extragonadal germ cell cancer syndrome (<50 years, lymph node involvement predominantly retroperitoneal or mediastinal), and women with predominant axillary lymph nodes and suspected breast cancer were excluded. Further inclusion criteria were ECOG PS <3, adequate bone marrow, renal and hepatic function (absolute neutrophil count >1000 *μ*l^−1^ and thrombocytes ⩾100 000 *μ*l^−1^, creatinine ⩽2 × UNL, bilirubine ⩽2 × UNL, AST/ALT ⩽5 × UNL), no prior chemotherapy, and the absence of brain metastasis or severe comorbidity. Age had to be within 18–80 years. Pregnant women were also excluded.

After written informed consent was obtained, patients were randomised to either arm A, paclitaxel 175 mg m^−2^ day 1 (d1) intravenously (3 h) and carboplatin AUC=5 mg ml^−1^ min^−1^ d1 i.v. (30 min), or to arm B, gemcitabine 1 g m^−2^ d1+8 i.v. (30 min) and vinorelbine 25 mg m^−2^ d1+8 i.v. (rapid short infusion). Cycles in both arms were repeated every 21 days. Response was assessed according to the WHO criteria every three cycles and every 3 months after end of treatment for at least 8 months. Patients with responsive or stable disease remained on therapy for a maximum of six cycles. Treatment after the completion of the planned six cycles of chemotherapy or after progression was at the discretion of the treating physician.

All toxicities were graded according to the NCI common toxicity criteria, version 3. Blood counts were checked weekly, clinical chemistry every three weeks. Dose reduction or delay of therapy was performed according to the following criteria: all patients received full dose of therapy on day 22 with leukocytes >2500 *μ*l^−1^ and thrombopenia grade ⩽1. In case of leukopenia ⩽2500 *μ*l^−1^ or thrombopenia grade ⩾2 therapy was postponed for 1 week, in case of grade ⩾3 leuko- or thrombopenia at day 22, treatment was reduced by 25% for all following cycles. For neurotoxicity CTC grade 2 treatment was delayed until recovery or reduced by 25% (at the discretion of the treating physician). In case of neurotoxicity grade 3 and 4 or any other CTC grade 4 toxicity (apart from alopecia and haematological toxicity) the patient was removed from the study leaving further treatment at the discretion of the treating physician.

The primary objective of the study was the composite endpoint ‘practicability’ of (1) treatment with at least two cycles of therapy (2) with a maximal delay of 1 week and (3) survival of ⩾8 months. This endpoint combines clinically relevant criteria concerning overall survival and the feasibility of the study therapy. It has been developed by the German Cancer Research Center and been used before in a randomised phase II trial (Manegold *et al*, 2005). It is in accordance to the EORTC guidelines to evaluate the response to treatment in solid tumours, which state that ‘measures of antitumor activity, other than tumour shrinkage, may allow investigation of cytostatic agents in phase II trials more appropriately’ (Therasse *et al*, 2000). The aim of the trial was to detect a practicability rate of >30% for each arm, respectively. The primary analyses were the two-sided 95% confidence intervals of the practicability rate for each arm. In each arm, assuming a practicability rate of 50%, 50 patients were to be evaluated to detect a practicability rate of >30% with 90% power at the 5% significance level. Secondary objectives were response rate, overall survival, progression-free survival and quality of life (measured by the EORTC QLQ C 30 questionnaire). Overall survival was calculated from the day of randomisation. Progression-free survival was measured from the day of randomisation until tumour progression or death was documented. Survival curves were constructed using the Kaplan–Meier method. Both arms were analysed separately, no adjustment for multiplicity was performed. Randomisation was accomplished using the permuted block randomisation with a block size or balancing interval of six patients. SAS, Version 9.1, was applied for all data analyses and the random number generation.

The study was approved by the local ethical committees and performed according to GCP standards. Source data verification was performed at every site throughout the study. The database was located at the KKS Mainz (Coordination Center for Clinical Studies, University of Mainz, Germany) which performed all descriptional and statistical analyses independently from the investigators. The trial was approved and registered by the German Cancer Society (Approval ‘Gutesiegel A’, clinical trial registration number 219, ‘Deutsches KrebsStudienRegister’).

## Results

**Figure 1:**
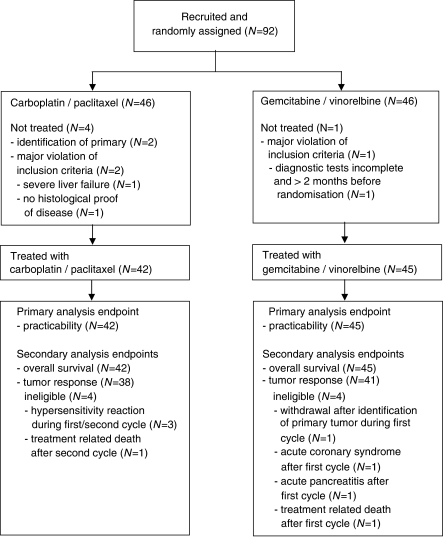
Trial profile, CONSORT diagram.

From September 2001 through June 2004, 92 patients were randomised in 25 German centers, contributing 1–16 patients each. Five patients had to be excluded from analyses due to major protocol violations before start of treatment: identification of the primary tumour (*N*=2), major protocol violations (*N*=3) (Figure 1). Of the remaining 87 eligible patients all were evaluable for the primary endpoint, 42 patients in arm A and 45 patients in arm B.

### Patient characteristics

Patient characteristics are given in Table 1. Both groups were well balanced with respect to gender, age, tumour burden, manifestation above or below the diaphragm, and LDH elevation. However, there was a slight tendency to a better ECOG performance score and to more lymph node involvement in arm A and a slight tendency to more liver involvement and pleural/peritoneal effusions in arm B (without reaching statistical significance). In the group of 16 patients with peritoneal involvement at presentation, 9 patients had ⩾3 and 5 patients had 2 involved sites. Only two patients had peritoneal involvement only (both female, arm B), ovarian cancer was ruled out in both. Overall, both arms represented a high-risk population of patients with CUP syndrome with a large tumour burden and a high proportion of patients with liver involvement.

### Delivery of scheduled chemotherapy

The median number of cycles was 5.5 in arm A and 3 in arm B, the mean number 4.3 and 3.6, respectively. In arm A 1–2 cycles were delivered to 10 patients, 3–5 cycles to 11 patients, and all planned 6 cycles to 21 patients. In arm B, 15 patients received ⩽2, 15 patients ⩽5 and 15 patients all 6 cycles. Mean dose intensity was ⩾90% for each drug throughout the study.

### Toxicity

Major toxicities are listed in Table 2. As expected, haematological toxicity was more common in arm B, whereas alopecia and hypersensitivity reactions (leading to withdrawal from the study in three patients) were more common in arm A. Febrile neutropenia was rare in both arms with a very low proportion of grade 3–4 infections. Three patients died from treatment-related toxicity. In arm B, one patient with extensive liver metastasis died of liver failure 3 days after the first application of chemotherapy, a second patient died of neutropenic sepsis 17 days after the first application of chemotherapy. In arm A, one patient was found dead at home 10 days after application of the second cycle of therapy, tumour progression combined with a major infection was the most probable cause.

### Efficacy

All patients were evaluable for the primary endpoint. The rate of practicability (as defined above) was 52.4% (22out of 42) in arm A and 42.2% (19 out of 45) in arm B, respectively (Table 3). Only in arm A the 95% CI was above the threshold of 30%, so this arm has proven to be practical as defined for the primary endpoint. Of 87, 79 patients were evaluable for response (38 out of 42 patients in arm A, and 41 out of 45 patients in arm B; Figure 1). There were no complete responders with either regimen. In the intention-to-treat analysis the overall response rate was 23.8% in arm A and 20.0% in arm B, respectively (Table 3).

**Figure 2:**
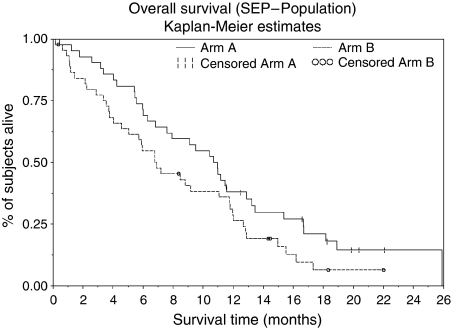
Overall survival.

**Figure 3:**
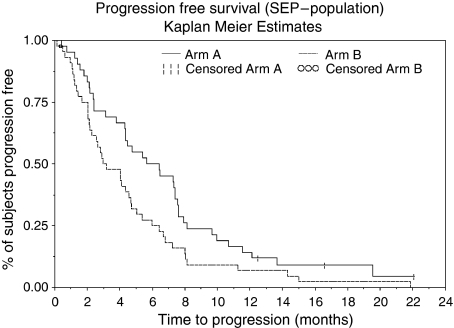
Progression-free survival.

The median progression-free survival was 6.1 (4.4–7.7) months in arm A and 3.2 (2.2–4.8) months in arm B, the median overall survival 11.0 (6.9–13.1) months and 7.0 (4.6–11.9) months for arm A and B, respectively. One-year survival rates were 38 (23–52)% and 29 (15–42)% for arms A and B (Figures 2 and 3). Data are mature; median follow-up in arm A was 18.5 months with 7 patients remaining at risk, arm B 14.6 months and 6 patients, respectively.

A Cox proportional hazard analysis failed to show a significant influence of ECOG performance status, elevation of LDH, number of involved sites, and liver involvement on survival.

## Discussion

Currently, a ‘gold standard’ of therapy in patients with adenocarcinoma or poorly differentiated CUP site (CUP syndrome) has not been established. To date, only eight randomised trials have been published in patients with CUP which have recruited 34–101 patients (Fizazi and Schmoll, 2006). Various chemotherapy regimen have shown limited activity in numerous phase II trials, resulting in response rates from 15 to 50% and a median survival of 4–11 months. Selection bias is a major problem in phase II trials in CUP because of the pronounced heterogeneity of the disease and the tendency of physicians to include patients who seem to be suited to the regimen under investigation. Thus, to obtain clinically meaningful results, randomisation and multicentricity are of major importance even in phase II trials.

On the basis of several phase II studies, platinum/taxane combinations are widely used in patients with CUP, yielding response rates of about 30% and a median survival of 7–11 months (Hainsworth *et al*, 1997; Briasoulis *et al*, 1998; Greco *et al*, 2002; Park *et al*, 2004; El-Rayes *et al*, 2005). Gemcitabine has also gained considerable attraction recently. As first-line regimen, gemcitabine combined with platinum or docetaxel achieved response rates of 31–55% with a median survival of 8–10 months (Culine *et al*, 2003; Pouessel *et al*, 2004; Pittman *et al*, 2006).

Our study was designed to investigate the widespread used combination of carboplatin and paclitaxel and the non-platin/non-taxane combination of gemcitabine and vinorelbine in patients with CUP syndrome in a randomised multicenter study. Dosage was chosen conservatively because of our concern of potentially avoidable toxicity in other trials with larger doses in a cohort of patients with palliative therapeutic intention. ‘Practicability’, a recently developed composite endpoint (Manegold *et al*, 2005) combining overall survival of at least 8 months and the application of at least 2 cycles of study treatment without significant delay was chosen to avoid endpoints of limited clinical relevance like overall response rate or progression-free survival in a palliative setting. Although a formal comparison of both treatment arms was not planned or performed, the study supports the importance of the widely used platinum/taxane schedule as this arm fulfilled the primary endpoint and reached a response rate of 23.8% and additional 40.5% with stable disease, resulting in 64.3% of patients with clinical benefit. The 1-year survival rate of 38%, the median survival of 11 months, and the progression-free survival of 6.1 months are in the upper expected range in this high-risk population of patients with CUP. Toxicity was tolerable and manageable. The gemcitabine/vinorelbine regimen also showed activity with a response rate of 20.0% and disease stabilisation in additional 33.3% (clinical benefit in 53.3% of patients). The main obstacle in this arm was the high early dropout rate with only 75.6% of patients receiving two or more cycles of therapy; partly due to early progression of disease. The 1-year survival rate was 29%, the median and progression-free survival 7.0 and 3.2 months, respectively. The rate of practicability was 42.2% with a 95% CI of 28–58%, including the 30% limit hypothesised; thus missing the aim of the study.

In contrast to other studies no complete remissions were observed in this study. This may be due to the inclusion of a high-risk population with a median age of 63 years, more than one involved site in 84%, liver involvement in 60%, and an ECOG performance score worse than 0 in 74% of patients. Although we applied a slightly lower dose of chemotherapy in the carboplatin/paclitaxel arm than comparable studies, the median survival of 11 months argues against a deleterious effect of the dosage applied. Moreover, these favourable results support the hypothesis that the addition of a third chemotherapeutic substance may not be beneficial in patients with CUP syndrome. In conclusion, the results of our trial argue strongly against the omission of platinum compounds and support the use of platinum/taxane combinations for the first-line treatment of CUP syndrome.

However, the efficacy of chemotherapy was modest with the vast majority of patients dying within 2 years which underlines the urgent need for an optimisation of treatment, for example by better characterisation of the tumour or validation of markers of response prediction like ERCC1 for platinum, RRM1 for gemcitabine, and tubulin mutation for taxane. Specific tumour cell profiles identified by gene expression microarrays may add to establish a working diagnosis in CUP (Ismael *et al*, 2006; Huebner *et al*, 2007; Bridgewater *et al*, 2008; Rosenfeld *et al*, 2008). The future will show how these techniques can be used to optimise and individualise patient care in CUP.

Another logical step is the addition of agents targeting the vascular endothelial growth factor or the epithelial growth factor receptor (EGFR) to an established chemotherapy regimen which has increased the treatment efficacy, for example in colorectal, breast, pancreatic, and NSCLC. Recent data have shown that for EGFR inhibitors – at least in colorectal and lung cancer – the mutational status of k-ras is a strong predictor of response efficacy (Linardou *et al*, 2008) which may apply to CUP, too. A recent pilot study combining bevacizumab and erlotinib in heterogeneous patients has shown considerable efficacy in CUP with a median overall survival of 7.4 months and 33% of patients alive at 1 year (Hainsworth *et al*, 2007). A high level of EGFR expression of 66% was observed in another study, with EGFR-expressing patients responding considerably better to platinum-based therapy (Massard *et al*, 2007). The German CUP study group has recently launched a randomised trial comparing the carboplatin/paclitaxel regimen used in this study with and without cetuximab to test the effectiveness of this EGFR inhibitor in patients with CUP.

## Figures and Tables

**Table 1:** Patient characteristics

	**Arm A, *n*=42 carboplatin/paclitaxel**	**Arm B, *n*=45 gemcitabine/vinorelbine**
Median age (range)	63 (31–77)	64 (46–75)
Male sex	23 (54.8%)	27 (60.0%)
		
*ECOG performance score*		
0	14 (33.3%)	9 (20.0%)
1	20 (47.6%)	25 (55.6%)
2	5 (11.9%)	10 (22–2%)
		
*No. of involved sites*		
1	6 (14.3%)	8 (17.8%)
2	10 (23.8%)	16 (35.6%)
3	18 (42.9%)	11 (24.4%)
>3	8 (19%)	10 (22.2%)
		
*Sites involved*		
Liver	23 (54.8%)	29 (64.4%)
Lung	14 (33.3%)	17 (37.8%)
Pleural effusion	3 (7.1%)	7 (15.6%)
Bones	8 (19.0%)	8 (17.8%)
Lymph nodes	27 (64.3%)	20 (44.4%)
Upper cervical	4 (9.5%)	2 (4.4%)
Supraclavicular	5 (11.9%)	4 (8.9%)
Axillary	4 (9.5%)	3 (6.7%)
Mediastinal	13 (31.0%)	10 (22.2%)
Abdominal	16 (38.1%)	11 (24.4%)
Peritoneum/ascites	5 (11.9%)	11 (24.4%)
Adrenal gland	4 (9.5%)	4 (8.9%)
		
Involvement above diaphragm	4 (9.5%)	3 (6.7%)
Below diaphragm	15 (35.7%)	17 (37.8%)
Above and below	23 (54.8%)	25 (55.6%)
		
LDH>upper normal limit	17 (40.5%)	18 (40.0%)

**Table 2:** Major toxicities

	**Arm A (% of patients)**			**Arm B (% of patients)**		
	**Total**	**Grade 3**	**Grade 4**	**Total**	**Grade 3**	**Grade 4**
Neutropenia	33.3	14.3	7.1	55.6	20.0	4.4
Thrombocytopenia	23.8	7.1	4.8	31.1	6.7	6.7
Fever/infection	26.2	2.4	2.4	46.7	8.9	2.2
Alopecia	73.8	16.7	2.4	22.2	2.2	0
Neurotoxicity	38.1	2.4	0	44.4	4.4	2.2
Nausea	52.4	0	0	62.2	11.1	0
Vomiting	23.8	0	0	31.1	0	2.2
Anorexia	23.8	9.5	0	37.8	15.6	0
Hypersensitivity	11.9	9.5	2.4	0	0	0

**Table 3:** Efficacy

	**Arm A**	**Arm B**
Practicable (95%-CI) as defined by	22 (52.4%; 36–68%)	19 (42.2%; 28–58%)
⩾2 cycles	39 (92.9%)	34 (75.6%)
Maximal delay 1 week	37 (88.1%)	34 (75.6%)
Survival ⩾8 months	25 (59.5%)	20 (44.4%)
		
Response	10 (23.8%)	9 (20.0%)
Partial response (PR)	6 (14.3%)	4 (8.9%)
PR, unconfirmed	4 (9.5%)	5 (11.1%)
Stable disease	17 (40.5%)	15 (33.3%)
Progression	11 (26.2%)	17 (37.8%)
Not assessable	4 (9.5%)	4 (8.9%)
